# Complete Management of a Mutilated Young Permanent Central Incisor

**DOI:** 10.5005/jp-journals-10005-1081

**Published:** 2011-04-15

**Authors:** Iqbal Musani, Varun Goyal, Asha Singh

**Affiliations:** 1 Professor, Department of Pedodontics and Preventive Dentistry, DY Patil Dental College and Hospital, Pune, Maharashtra, India; 2 Postgraduate Student, Department of Pedodontics and Preventive Dentistry, DY Patil Dental College and Hospital, Pune, Maharashtra, India; 3 Director, Postgraduate Studies, Department of Pedodontics and Preventive Dentistry, DY Patil Dental College and Hospital Pune, Maharashtra, India

**Keywords:** Anatomic post and core, Apexogenesis.

## Abstract

This case report throws light on treatment of immature apices through apexogenesis and an esthetic postobturation restoration of traumatized young permanent central incisor using a relatively newer methodology of anatomic posts, i.e. shaping the post to the root anatomy. The authors would also like to underline the significance of rubber dam isolation for more predictable outcomes. The new method of anatomic post is simple, viable, practical, and less time consuming than thought.

## INTRODUCTION

Immature teeth require some form of endodontic intervention due to extensive caries or traumatic injury. When such a clinical situation presents itself, an assessment of the pulpal status and the degree of tooth development must be made in order to develop an appropriate treatment plan that is conducive to long-term tooth retention.^1-4^

Tooth retention also depends on coronal preservation of what is remaining and replacement of lost tooth structure. This article throws light towards treatment of immature apices through apexogenesis and an esthetic coronal restoration of traumatized young permanent central incisor using a relatively newer methodology of anatomic posts, i.e. shaping the post to the root anatomy.

Literature suggests that depending on the vitality of the affected pulp, there are various treatment modalities to treat a young permanent tooth:^1,15^

1. Revascularization
2. Apexogenesis—Ca(OH)_2_
3. Apexification:
  - Single visit—MTA
  - Multiple visit—Ca(OH)_2_
4. Customized cone technique using roll cone
5. Periapical surgery.

Most endodontically treated teeth require intraradicular devices for restoring teeth to optimum health and function. These devices vary from conventional custom cast post and core to prefabricated post systems.

Traditionally used custom cast post and core are rigid metal post system that resist lateral forces without distortion but result in undue stresses to less rigid dentin causing potential root fracture. But fiber post system flexes under lateral loading and prevents undue stress.

## CASE REPORT

**Fig. 1:**
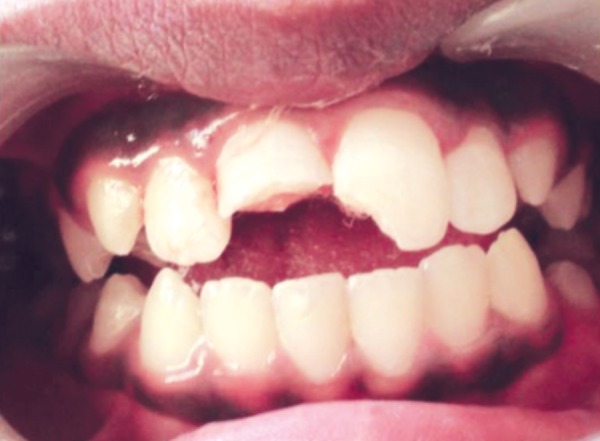
Preoperative photograph when the patient first reported

A 11-year-old boy reported to Department of Pediatric and Preventive Dentistry at Dr DY Patil Dental College and Hospital Pimpri, Pune with a chief complaint of fractured right and left incisor. The patient gave a history of trauma at age 10 (Fig. 1).

Examination revealed a complicated crown fracture with 11 and uncomplicated crown fracture with 21. Cold vitality test elicited a painful and lingering response with 11 and radiographically it was evident that roots had incomplete apical root end formation with wide open apices in 11 and 21.

A diagnosis of11 was irreversible pulpitis. It was deemed to be highly desirable in this case to leave radicular pulp tissue vital in order to allow for continued root development and apical closure—apexogenesis.

## TREATMENT

After administration of local anesthesia, tooth number 11 was isolated with rubber dam and coronal access was gained and 2 to 3 mm of inflamed coronal pulp tissue was removed using diamond burs under copious irrigation. Time was allowed for bleeding to arrest and again the pulp chamber was gently rinsed to remove any blood clot from the surface of remaining pulp tissue.

**Fig. 2:**
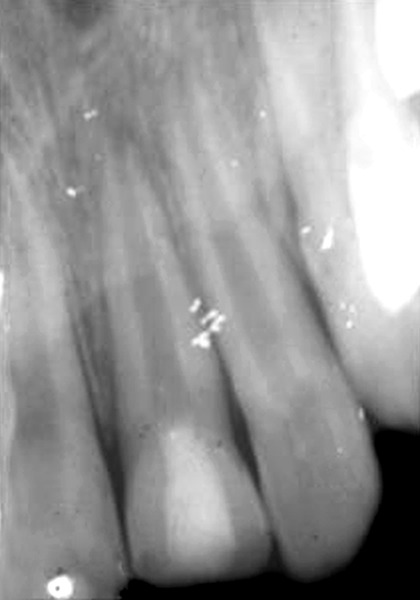
Radiograph immediately after pulpotomy

Once hemostasis was achieved, a thick mix of Ca(OH)_2_ paste (Dycal) was placed over exposed pulp tissue and slightly condensed. IRM was placed and a glass ionomer restoration was used as a coronal semipermanent restoration (Fig. 2).

**Fig. 3:**
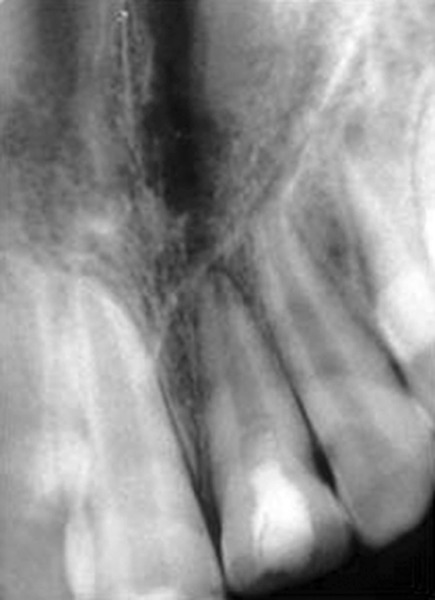
Three months after pulpotomy

**Fig. 4:**
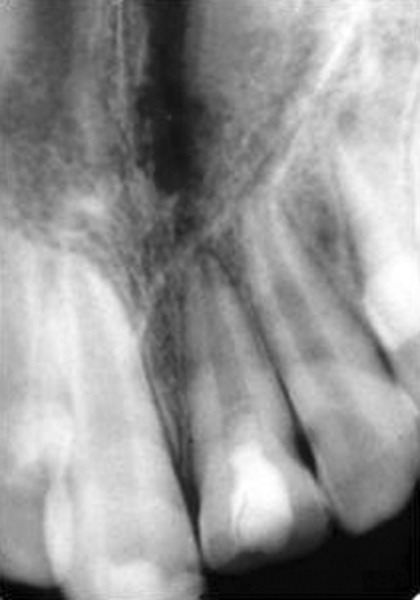
Six months after pulpotomy

The patient was monitored clinically and radio-graphically every month to assess for continued root development and for signs and symptoms of pulp deterioration namely necrosis, infection, root resorption or periradicular pathosis (Figs 3 and 4). Tooth 21 was restored with composite material.

## FOLLOW-UP VISIT

At 9 months recall examination, radiograph revealed complete root development. It was decided to perform a complete pulpectomy for the said tooth.

**Fig. 5:**
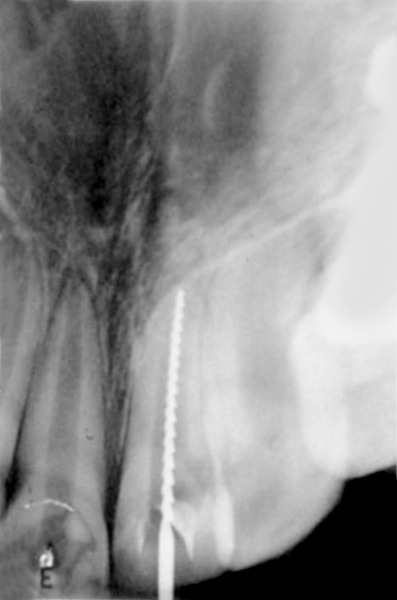
Nine months later access opening was done

**Fig. 6:**
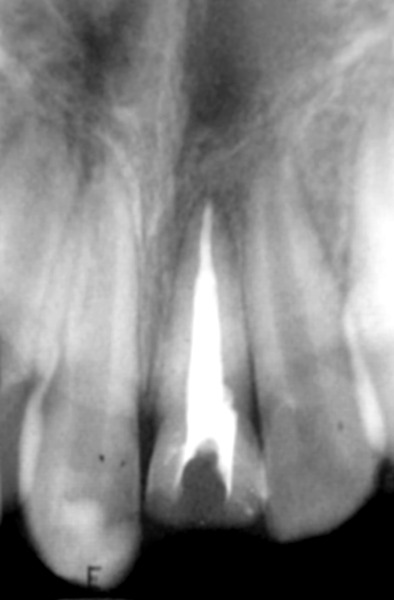
Final obturation

**Fig. 7:**
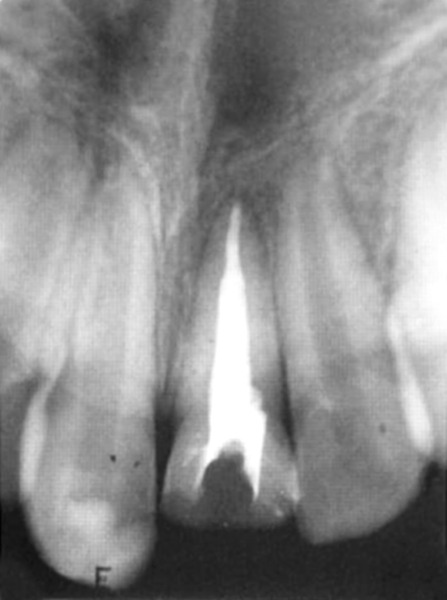
Four weeks after obturation

Conventional endodontic therapy was performed (Figs 5 to 7). The root canal anatomy after endodontic treatment was such that no prefabricated post could satisfactorily adapt to remaining internal dentinal walls of the root canal. In addition, the amount of remaining dentin thickness was less. It was anticipated that in these clinical situations, the prefabricated post cementation may not satisfactorily dissipate the stresses, which could lead to vertical root fracture and the eventual loss of the tooth. Since the post design materials used and post space preparation have a significant influence on vertical fracture, a post with a Young’s modulus approaching that of dentin was more desirable in this case, as stresses transmitted on loading of the post will decrease, thus reducing the risk of a root fracture.^2,3^

The decision was made to use an anatomic fibre post for tooth 11.

**Fig. 8:**
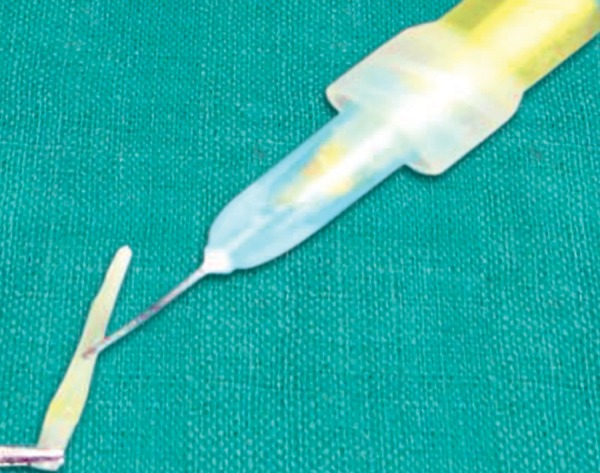
Pretreatment of the post with HF acid

**Fig. 9:**
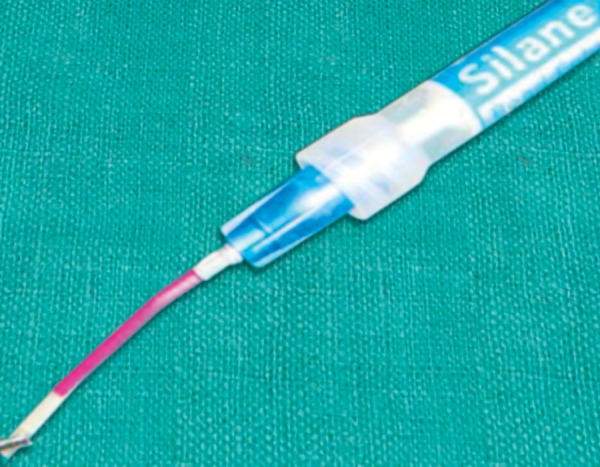
Treatment with coupling agent

After obtaining the post room, the prepared root canal was then lubricated with glycerin. A translucent fiber post was selected (DT light post) and pretreated with hydrofloric acid (Fig. 8) followed by the application of coupling agent to facilitate the bonding between composite and fiber post material (Fig. 9).

**Fig. 10:**
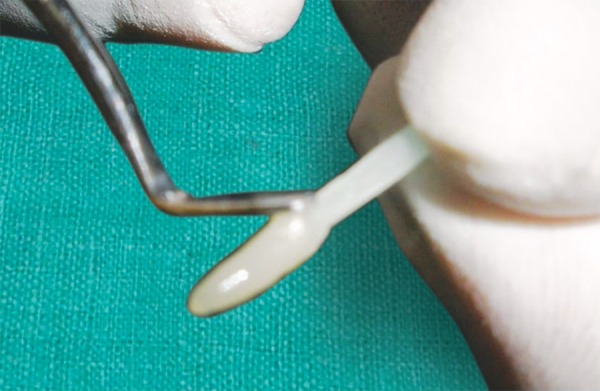
Some more melted composite being applied to the post for customization

**Fig. 11:**
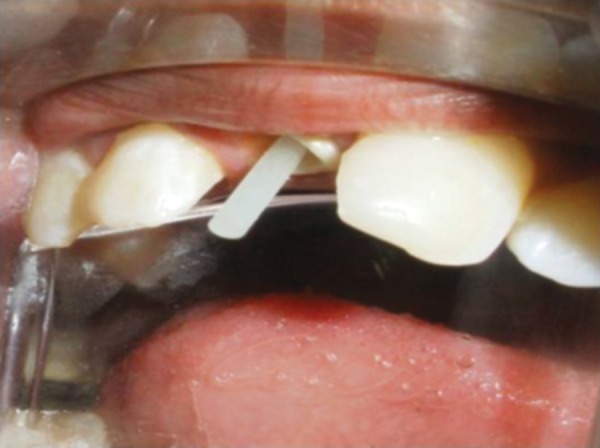
Custom post in place

**Fig. 12:**
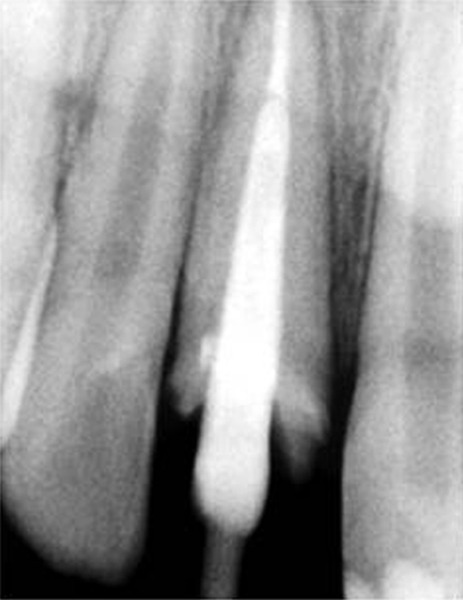
Radiographic confirmation of the custom post

Resin composite (Z-350 3M ESPE) was warmed at 40°C for 2 minutes and then coated over the post and inserted into the canal, adapting it precisely to replicate the canal anatomy (Fig. 10).^6,7^ This was light cured for 20 seconds. The anatomic post was tried again, in order to ensure easy insertion, without any interference. The luting of this anatomic post assembly was performed similar to that recommended for a conventional translucent post. The root canal walls were etched with 37% phosphoric acid for 15 seconds, washed with a water syringe and gently dried. Four to five coats of fifth generation bonding systems (3 M ESPE) were applied into the root canal with microbrush applicator. Rely-X (3M ESPE) a dual cure resin cement was used for luting (Figs 11 and 12).

Immediately after post cementation, a core build up with composite resin and crown preparation was done to receive a temporary acrylic crown. The patient was then referred to the department of orthodontics for correction of bilateral posterior cross bite and anterior open bite. A permanent full ceramic crown would be planned postorthodontic treatment.

## DISCUSSION

*Apexogenesis* is a vital pulp therapy procedure performed to encourage continued physiological development and formation of the root end. Traditionally, this has implied removal of the coronal portion of the pulp. However, the depth to which the tissue is removed should be determined by clinical judgment. Only the inflamed tissue should be removed.

The goals of apexogenesis, as stated by Webber are as follows:

1. Sustaining a viable Hertwig’s sheath, thus allowing continued development of root length for a more favorable crown-to-root ratio.
2. Maintaining pulpal vitality, thus allowing the remaining odontoblasts to lay down dentine, producing a thicker root and decreasing the chance of root fracture.
3. Promoting root end closure, thus creating a natural apical constriction for root canal filling.
4. Generating a dentinal bridge at the site of the pulpotomy. While the bridging is not essential for the success of the procedure, it does suggest that the pulp has maintained its vitality.

**Fig. 13:**
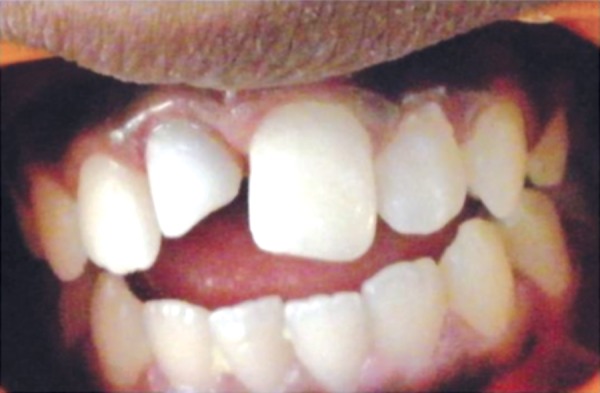
During composite core build up

**Fig. 14:**
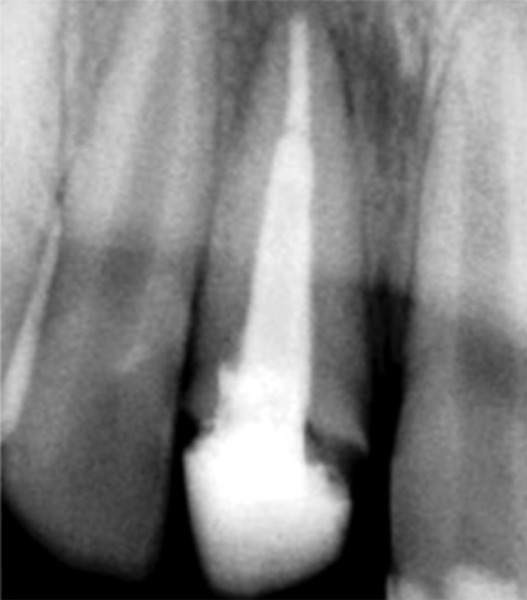
Radiographic confirmation during core build up

**Fig. 15:**
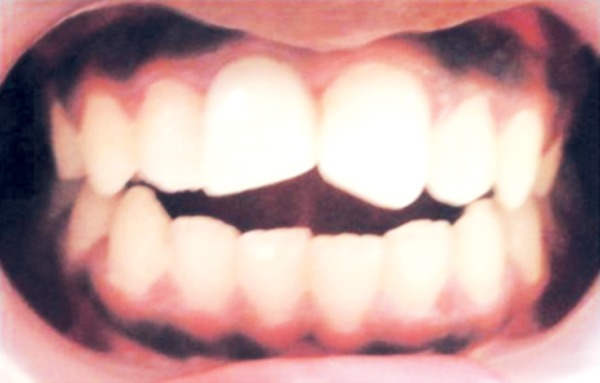
Immediately after composite core build up 11 and 21

The total time for achievement of the goals of the apexogenesis ranges between 1 and 2 years depending on the degree of tooth development at the time of the procedure.^2^

In the present case, success was achieved following strict asepsis protocol throughout all the clinical steps.

*Post and core* is done to replace missing coronal tooth structure to provide the required retention and resistance from for the final restoration. Post are of two basic types; readymade and custom-made and may be either resin or metal posts.

Use of metal post has declined, as they let to unsalvageable fractures due to a wedging effect and there metal shadow is difficult to mask. Resin posts are esthetic compatible and, as they are bonded to root dentin, the outcome is a better stress distribution over the root surface (Freedman 2001).^9^

The evolution in the technology has enabled manufacturers today to provide fiber posts, which besides offering superior esthetic and mechanical properties, are radiopaque and available in variety of shapes. Fiber posts are meant for the better adaptation of the post to the canal anatomy, thus minimizing the amount of residual root structure that has to be sacrificed in order to obtain proper post fit. This trend towards more conservative root preparations for the post adaptations has been possible because of contemporary progress in the field of materials and technique for bonding, which has made adhesion to the root canal wall more predictable.^4,5,8^ It is likely that a further significant improvement in the fiber post adaptation and retention will be achieved with so called anatomic post. This is a translucent fiber post covered by a layer of light cure resin composite, which allows for an individual, anatomic shaping of the post through its insertion into the canal with the aim of achieving better fit than is possible with any prefabricated post.^10^ As the result of its precise adaptation to the root canal shape, the individual post is surrounded by a thin and uniform layer of resin cement, which creates an ideal condition for post retention. This procedure of ‘individualizing’ the post through the resin layer is advisable in the canal exhibiting a reduced amount of residual root surface after endodontic treatment.^3,9^ This composite reinforced post is the procedure of choice in cases of wide canals with less dentin.^11^

## CONCLUSION

Apexogenesis is a highly successful procedure if conducted under strict asepsis and with the correct clinical diagnosis. Anatomic post can be used for reconstructing endodontically treated teeth, when there is loss of tooth substance at the coronal level as well as when the root anatomy of endodontical teeth is not circular and the root canal walls have reduced dentine thickness. With only 5 minutes of additional clinical time, it is possible to obtain a well-fitting anatomic post, which is superior to any other prefabricated posts. Anatomic post is easy to fabricate, time saving and economical. These posts are esthetically acceptable, bonded to root dentine, thus behaving as a monobloc for better stress distribution.
